# MOZAIC: compound growth via *in silico* reactions and global optimization using Conformational Space Annealing

**DOI:** 10.1093/bioinformatics/btag595

**Published:** 2026-08-07

**Authors:** Jinhyeok Yoo, Woong-Hee Shin

**Affiliations:** Department of Biomedical Informatics, Korea University College of Medicine, Seoul 02708, Republic of Korea; Department of Biomedical Informatics, Korea University College of Medicine, Seoul 02708, Republic of Korea; Arontier Co, Seoul 06775, Republic of Korea

## Abstract

**Motivation:**

Fragment-based drug discovery (FBDD) efficiently explores chemical space by combining small molecular fragments. Advances in computational methods are accelerating the development of algorithm- and AI-based approaches in FBDD. However, it should be noted that certain methods do not provide synthetic pathways to obtain the proposed compounds. Consequently, these molecules might not be synthesized easily.

**Results:**

We present MOZAIC, a reaction-based fragment-growing framework that combines in silico reactions with Conformational Space Annealing for global molecular optimization. MOZAIC generates compounds through SMARTS-defined organic reactions, preserving reaction histories and providing putative synthetic routes. Across benchmark targets, MOZAIC produced chemically diverse molecules with balanced improvements in predicted binding affinity, drug-likeness, and synthetic accessibility. Compared with existing fragment-growing and generative approaches, MOZAIC achieved broad scaffold coverage while maintaining target-directed optimization. Its modular objective function also enabled alternative design goals, such as improving predicted solubility while maintaining binding affinity.

**Availability and implementation:**

MOZAIC is available at https://github.com/kucm-lsbi/MOZAIC.

## Graphical Abstract

**Figure:**
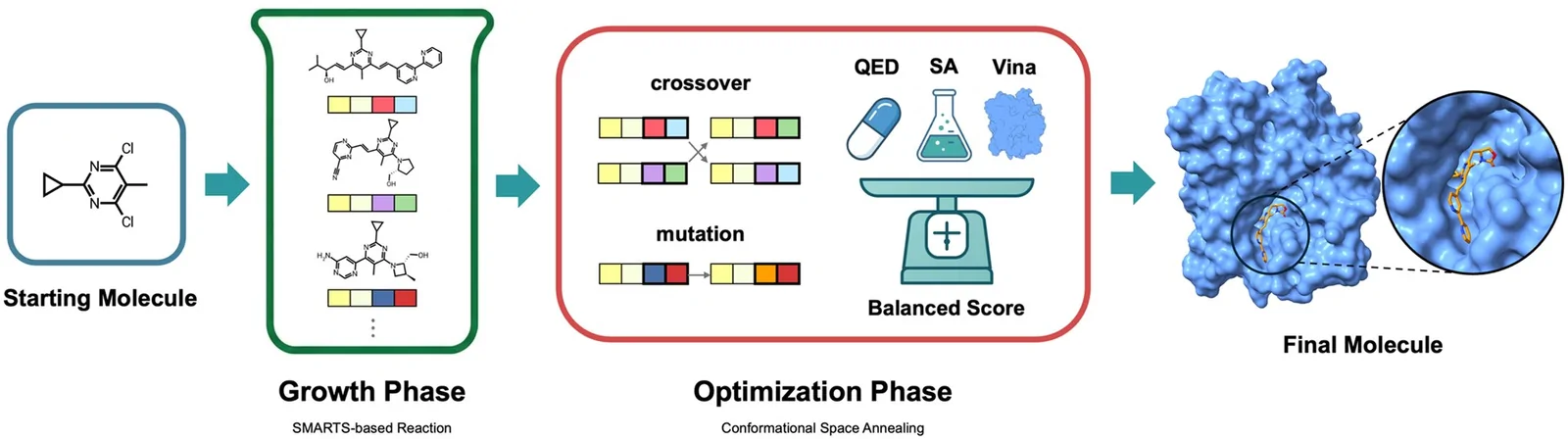

## 1 Introduction

Fragment-based drug discovery (FBDD) has been successfully applied in drug discovery by exploring potent binding fragments to the subpocket of a receptor (Khedkar *et al*. 2025, Santos and da Silveira 2023, Xu and Kang 2025). Because fragments have simple structures and favorable drug-like properties, they often yield higher hit rates than high-throughput screening (Hajduk and Greer 2007). Their combinatorial diversity also enables cost- and time-efficient exploration of chemical space using relatively small libraries. FBDD strategies for molecular optimization are generally categorized into fragment growing, merging, and linking (Yoo *et al*. 2025). Among these, fragment growing expands an initial fragment to improve molecular properties and binding interactions, guided by the binding pose and the stability of growth.

Recent computational FBDD methods have increasingly addressed multi-objective optimization, including drug-likeness and docking scores, using algorithmic or AI-driven approaches (Angelo *et al*. 2023). AutoGrow4 (Spiegel and Durrant 2020) applies a genetic algorithm to generate compounds from initial fragments, followed by filtering and selection based on docking scores and diversity. CReM-dock (Minibaeva *et al.* 2026) connects filtered fragments and evaluates generated molecules using AutoDock Vina (Trott and Olson 2010) or GNINA (McNutt *et al*. 2025) within a genetic algorithm. FRAME (Powers *et al*. 2023) predicts growth directions and fragments using SE(3)-equivariant models, whereas ReBADD-SE (Choi *et al*. 2023) applies reinforcement learning with SELFIES (Krenn *et al*. 2022) to guide fragment growth according to target activity and drug-like properties. Although these methods have advanced computational FBDD, generated molecules may be biased toward local optima or training-set distributions, and their synthesis might be challenging (van den Broek *et al*. 2025).

To address the challenges, we propose MOZAIC (molecule optimization via AutoDock Vina, in Silico fragment reaction, and conformational space annealing), a fragment-growing framework that combines in silico reactions with Conformational Space Annealing (CSA) (Joung *et al*. 2018). MOZAIC modifies a starting molecule using SMARTS-defined reaction rules based on real organic reactions. SMARTS (https://www.daylight.com) is an extension of SMILES to specify molecular substructure patterns. The reaction rules are defined by identifying substructures in the reactants and transforming them to generate the products. By identifying reactive motifs and recording reaction histories, MOZAIC provides putative synthetic routes for generated compounds. CSA, a powerful population-based global optimization algorithm, has been successfully applied to diverse biological and chemical optimization problems, including protein-ligand docking, cryo-EM map fitting, molecular generation, and reaction-pathway exploration (Shin and Seok 2012, Shin *et al*. 2013, Lee *et al*. 2017, Kwon and Lee 2021, Park *et al*. 2023). Recently, CSearch (Kim *et al*. 2024) adapted the CSA algorithm to chemical-space optimization using BRICS (Degen *et al*. 2008) retrosynthesis rules and a graph neural network surrogate trained to approximate docking energies. In contrast, MOZAIC uses SMARTS-based forward reactions to append fragments while preserving the core scaffold and maintaining traceable reaction histories.

MOZAIC evaluates candidate molecules using a flexible multi-objective function composed of AutoDock Vina score, synthetic accessibility (SA) (Ertl and Schuffenhauer 2009) score, and drug-likeness score (QED) (Bickerton *et al*. 2012). This design enables simultaneous optimization of predicted binding affinity, synthesizability, and drug-likeness. Because the objective components are modular, they can also be replaced to target other molecular properties, such as solubility. By integrating reaction-based fragment growing, traceable synthesis information, and CSA-driven global optimization, MOZAIC provides a practical framework for fragment-to-lead and lead optimization in drug discovery.

## 2 Materials and methods

### 2.1 Overview of MOZAIC

**Figure 1:**
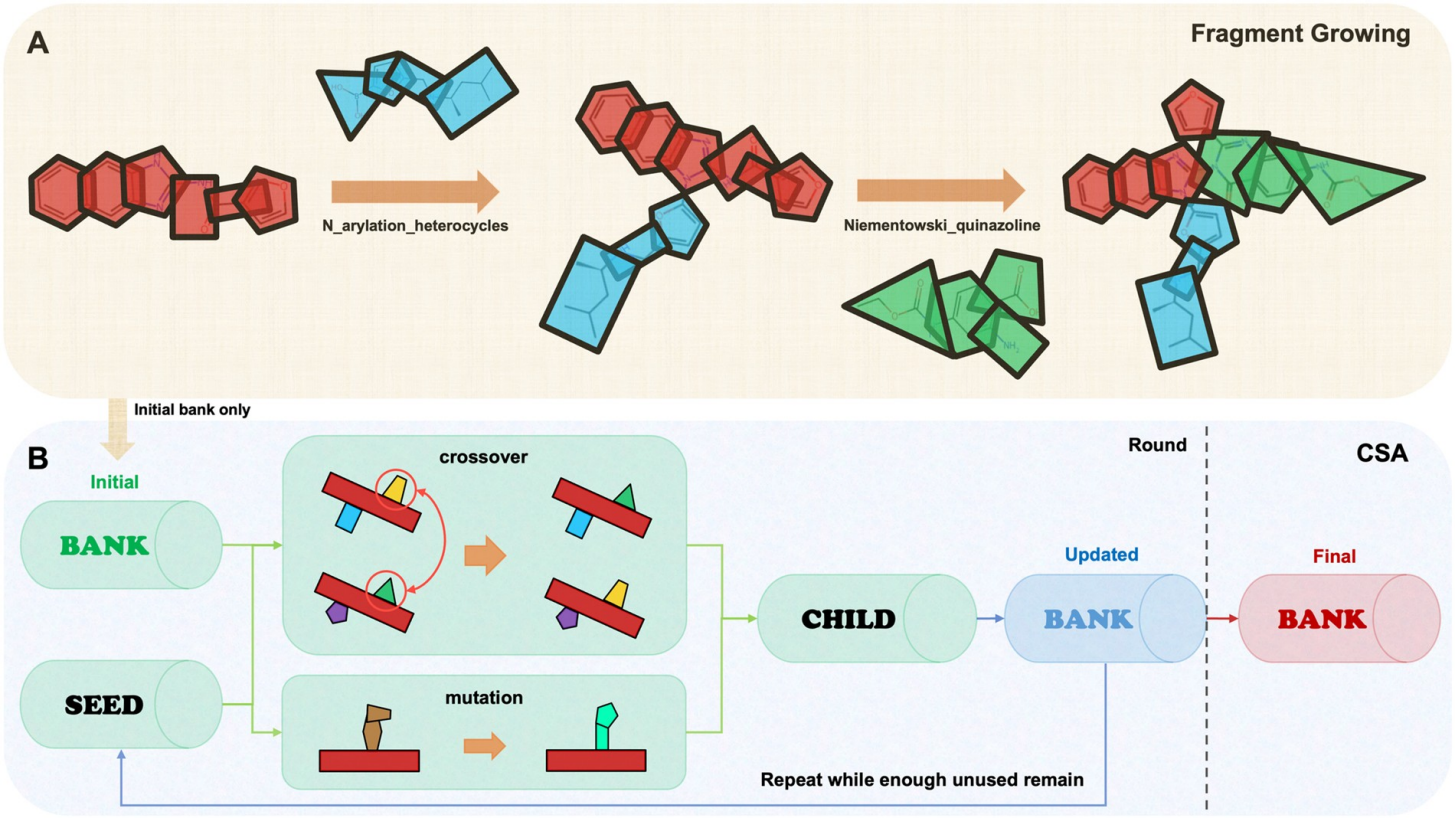
Overview of the MOZAIC framework. (A) Fragment growing according to in Silico reaction rules. Molecules are generated by adding fragments (blue and green) to the input compound (red) to construct the initial pool of molecules. The initial bank is constructed by selecting compounds as diverse as possible. (B) Optimization using CSA. From the current bank, seeds and partners are selected randomly to generate children via crossover and/or mutation. One round of CSA ends when the number of unused molecules available as seeds falls below a predefined threshold. The final bank is obtained after three rounds of CSA. The arrow from (A) to (B) indicates that fragment growing is used only to construct the initial bank.

MOZAIC is a fragment-growing molecular optimization framework designed to generate target-specific, synthesizable compounds with drug-like properties. As shown in Fig. 1, the pipeline consists of two phases: initial bank construction and CSA-based optimization. First, SMARTS-based reaction rules are applied to attach fragments to a starting molecule, generating an initial bank of candidate solutions (Fig. 1A). In CSA terminology, the bank represents the population of solutions.

The input consists of a three-dimensional protein structure, binding-site residue indices, and a SMILES string for the starting molecule. Each generated solution stores the starting functional group, reaction name and position, added fragment, and product SMILES string. Molecules are evaluated using an objective function combining QED, SA score, and AutoDock Vina score to balance drug-likeness, synthesizability, and predicted binding affinity.

In the CSA phase (Fig. 1B), seed molecules are randomly selected from the current bank and modified through crossover or mutation using partner molecules from the current bank or the initial bank. The resulting children are compared with existing bank members based on molecular distance and objective score, and the bank is updated accordingly. This optimization cycle is repeated for three CSA rounds to obtain the final bank. Fragment growing is used only during initial bank construction; subsequent CSA operations recombine or mutate reaction histories already present in the initial or current bank. The details of the CSA iteration will be given in the next subsection.

### 2.2 Application of CSA to molecule optimization by fragment-growing

#### 2.2.1 Objective function for evaluating the fitness of a molecule

MOZAIC evaluated each molecule using a three-component objective function that balances drug-likeness, synthetic accessibility, and predicted binding affinity. Drug-likeness is measured by QED, which ranges from 0 to 1. Synthetic accessibility is assessed by the SA score, where lower values indicate easier synthesis. Binding affinity is estimated by the AutoDock Vina score, where lower DG values indicate stronger predicted binding.

Because these metrics have different scales and directions, the SA and Vina scores are normalized using Equations (1) and (2). The normalized values are then combined with QED to produce a final objective score ranging from 0, the worst, to 3, the best.


(1) $${\mathrm{SA}}_{\mathrm{norm}}=1-\frac{\mathrm{SA}-1}{9}$$

(2) $${\mathrm{Vina}}_{\mathrm{norm}}=\frac{{\mathrm{Vina}-\mathrm{Vina}}_{\min}}{{\mathrm{Vina}}_{\max}-{\mathrm{Vina}}_{\min}}$$

QED and SA scores are calculated using RDKit (http://www.rdkit.org), while binding affinities and poses are obtained from AutoDock Vina. The input protein structure is prepared by removing hetero-molecules, including water. A cubic docking box of 20 Å per side is centered on the geometric center of the selected binding-site residues. For each molecule, AutoDock Vina generates ten poses, and the lowest docking score is used in the objective function.

#### 2.2.2 Initial bank construction by applying in Silico reaction rules

The performance of CSA depends on the diversity and quality of the initial bank (Shin *et al*. 2013). To construct this bank, MOZAIC first generates a candidate pool of 1000 molecules by adding fragments to the starting molecule using SMARTS-defined in silico reaction rules. A total of 104 reactions from AutoClickChem (Durrant and McCammon 2012), Robust Rxn database (Hartenfeller *et al*. 2011), and ChemoDOTS set (Hoffer *et al*. 2024) are used. For a given input molecule, MOZAIC identifies atoms or bonds capable of participating in these reactions, selects compatible fragment subsets, and randomly incorporates fragments from the fragment library. To preserve the original scaffold, the maximum common substructure (MCS) between the initial and current compounds is identified. Initial functional-group atom indices are mapped onto the current compounds so that the reactions occur only at the original reactive sites. The reaction histories, including the step, reaction position, and attached fragment, are recorded for later crossover and mutation. Each reaction also has a 10% chance of being skipped to increase molecular diversity.

The RDKit MaxMinPicker algorithm (Ashton *et al*. 2002) was used to select 60 diverse compounds from the candidate pool for initial-bank construction. Starting from a randomly selected molecule, MaxMinPicker iteratively selects the compound whose minimum distance to the previously selected set is maximal. Molecules were represented by 2048-bit Extended Connectivity Fingerprints (ECFP) (Rogers and Hahn 2010) fingerprints with radius 4, and molecular distance was calculated as 1 − *T_c_*, where *T_c_* is the Tanimoto similarity. Tanimoto similarity is defined as *T_c_ = c/(a + b–c)*; *a* and *b* denote the number of bits set in the fingerprints of two molecules, and *c* denotes the number of common bits. After selecting the 60 compounds, half of the average pairwise distance, *D_avg_*/2, was used as the initial *D_cut_* value.

#### 2.2.3 New molecule generation, bank update, and CSA iteration

At each CSA iteration, MOZAIC generates new molecules, or children, using crossover and mutation. Thirty seed molecules are randomly selected from the current bank. For crossover, each seed is paired with two partner molecules, one from the initial bank and one from the current bank. The initial bank helps preserve sampling diversity, whereas the current bank promotes exploration of high-scoring regions. If a seed and partner share the same reaction functional group, the seed reaction information, including the attached fragment, is replaced with that of the partner. Mutation randomly reselects reaction components based on the atom used for the reaction. In total, 90 children are generated per iteration: 60 by crossover and 30 by mutation.

Each child is compared with the current bank using molecular distance, *D*, and the objective score. If the child lies within *D_cut_* of any current bank member, it replaces the closest member only if it has a higher score. If it lies farther than *D_cut_* from all current bank members, it replaces the lowest-scoring bank member. *D_cut_* is gradually reduced from *D_avg_*/2 to *D_avg_*/5. Thus, *D_cut_* prevents accepted solutions from collapsing into similar structures and preserves bank diversity. Unlike conventional genetic algorithms, which often select offspring mainly by fitness and may converge prematurely, CSA accepts children based on both objective score and distance from existing bank members. As *D_cut_* decreases, the search shifts from broad exploration to focused optimization, reducing the risk of convergence to local minima.

Seed molecules used in an iteration are marked as used, and later seeds are selected from the unused pool. A CSA round ends when fewer than 30 unused molecules remain. The process is repeated for three rounds to generate the final bank.

#### 2.2.4 Final bank analysis and MOZAIC output

MOZAIC provides the optimized final-bank molecules and their predicted docking poses with the target receptor. The resulting protein–ligand complex structures can be further analyzed with PLIP (Salentin *et al*. 2015) to identify interactions such as hydrogen bonds, hydrophobic contacts, salt bridges, π-stacking, π-cation interactions, halogen bonds, water bridges, and metal complexes by checking the geometry formed by the atoms. The final output includes information for both initial- and final-bank compounds, including SMILES strings, objective scores, reaction details, and added fragments, together with target–ligand complexes for 3D inspection and interaction profiling.

### 2.3 Fragment library construction

Because 89 of the 104 reactions are bimolecular, MOZAIC requires reaction-specific fragment libraries. As shown in Supplementary Data Fig. S1, available as supplementary data at *Bioinformatics* online, fragments were prepared from the ZINC20 library, accessed July 2025 (Irwin *et al*. 2020). Compounds violating the Rule of Three (Jhoti *et al*. 2013) were removed, and the remaining compounds were classified according to the SMARTS patterns of the reaction rules. Considering the functional groups used in the reactions, 103 fragment subsets were constructed. For reaction patterns matching more than 10 000 fragments, the fragments were sorted by molecular weight, and the 10 000 lowest-weight fragments were retained. The final fragment library contained 893 675 fragments.

### 2.4 Benchmark set for MOZAIC

Three benchmark sets were prepared to evaluate MOZAIC. First, two FBDD examples were selected from studies by Woodhead and colleagues (Woodhead *et al*. 2024, Holvey *et al*. 2025). Phosphodiesterase 10A (PDE10A), a cyclic nucleotide phosphodiesterase involved in cAMP/cGMP signaling (Amin *et al*. 2021), complexed with MK-8189 (PDB ID: 8DI4), was used to assess the effect of initial-bank quality. In addition, tRNA-(N^1^G37) methyltransferase (TrmD), an essential bacterial tRNA-modifying enzyme investigated as an antibacterial target (Hill *et al*. 2013), complexed with BDBM50613275 (PDB ID: 8APW), was used for comparison with CReM-dock and REINVENT4 (Loeffler *et al*. 2024). The selection was based on the number of functional groups in the initial molecule and the existence of a fully grown ligand complexed with the target protein in the PDB. For CReM-dock, AutoDock Vina parameters were kept identical to MOZAIC, the chembl22_sa2_hac12.db fragment database was used, and nclust and max_replacements were set to 2 and 100, respectively. For REINVENT4, an open-source molecular design framework, the R-group replacement mode was used with an objective function combining QED and normalized SA score; 100 000 molecules were generated, and the top 60 molecules were docked to the TrmD via AutoDock Vina.

To compare the diversity, Bemis-Murcko scaffold (BMS) (Bemis and Murcko 1996) was extracted from the outputs of the programs. From the two-dimensional structure of a compound, the scaffold extracts the core framework by keeping rings, linker atoms connecting rings, and the bonds between the atoms. In contrast, terminal side chains, substituents attached to the core, and peripheral decorations are removed. A scaled Shannon entropy (SSE) of BMS is computed as Equation (3) to quantify diversity (Medina-Franco *et al*. 2009).


(3) $$\mathrm{SSE}=-\frac{\sum_{i=1}^{K}p_{i}\log p_{i}}{\log\;K}$$

*p_i_* is the fraction of molecules assigned to the *i-*th scaffold, and *K* is the number of unique scaffolds. The SSE ranges from 0 (all compounds share a single scaffold) to 1 (compounds are uniformly distributed across all scaffolds).

Second, MOZAIC was tested under receptor-structure variation using beta-2 adrenergic receptor (ADRB2). Active ChEMBL (Gaulton *et al*. 2012) ligands with pK_d_ or pK_i_ > 7 were clustered, and the largest cluster whose MCS contained at least two reaction-compatible functional groups was used to define the starting molecule. The overall process for starting fragment selection is illustrated in Supplementary Data Figure S2, available as supplementary data at *Bioinformatics* online. The carazolol-bound ADRB2 structure (PDB ID: 2RH1, resolution: 2.40 Å) and an AlphaFold3 (AF3) (Abramson *et al*. 2024) predicted structure aligned to the crystal structure, using UCSF Chimera MatchMaker (Meng *et al*. 2006), were used as receptors. The RMSD between the receptors was 0.746 Å. The crystal structure was previously studied in structure-based docking and virtual screening studies (Kolb *et al*. 2009). The Tanimoto similarity between carazolol and the starting compound is 0.091. Binding-site residues in the AF3 model were defined from the residues within 5 Å of carazolol in the crystal structure. After optimizing the molecule, the top molecules were compared to the known active ligands. The local confidence of these residues was quantified by atom-averaged pLDDT scores, yielding a mean binding site pLDDT of 87.41.

Third, the flexibility of the MOZAIC objective function was examined using peroxisome proliferator-activated receptor gamma (PPARγ). The objective function, such as QED, can be easily substituted with alternative molecular descriptors, thereby providing flexibility in the optimization of multi-objectives in molecular design. To optimize solubility while maintaining binding affinity, QED and SA were replaced to the predicted solubility with ESOL (Delaney 2004) and docking score. ESOL is a linear regression model to predict logS of a molecule using Equation (4).


(4) logS=0.16−0.63·logP−0.0062·MW+0.066·Rot−0.74·Aromatic

where *MW*, *Rot*, and *Aromatic* represent molecular weight, the number of rotatable bonds, and the proportion of aromatic atoms, respectively.

PPARγ belongs to the nuclear receptor family, and acts as a key metabolic switch for regulation of adipocyte differentiation and glucose homeostasis (Houseknecht *et al*. 2002). For nuclear receptors, many potent ligands are relatively lipophilic (Zhang *et al*. 2015); therefore, improving aqueous solubility while maintaining or even improving affinity is critical for oral exposure, formulation flexibility, and predictable pharmacokinetics.

CHEMBL375270 (EC_50_: 2.0 nM), the lowest-molecular-weight ChEMBL compound with pChEMBL > 8 was selected as the initial molecule, and PPARγ structure 6MS7 (resolution: 1.43 Å) was used as the PPARγ receptor.

## 3 Results and discussion

### 3.1 Applying MOZAIC to optimize PDE10A hit compound, comparing initial bank construction methods, and investigating hyperparameters

#### 3.1.1 Optimizing PDE10A hit compound using MOZAIC

To validate MOZAIC, PDE10A was selected as the first benchmark. The starting molecule, 4,6-dichloro-2-cyclopropyl-5-methylpyrimidine, has a *K_i_* of 8.7 mM and was experimentally optimized to MK-8189 (Layton *et al*. 2023), a selective PDE10A inhibitor with *K_i_* = 0.029 nM. MOZAIC was applied to the co-crystallized PDE10A-MK-8189 structure.

**Figure 2:**
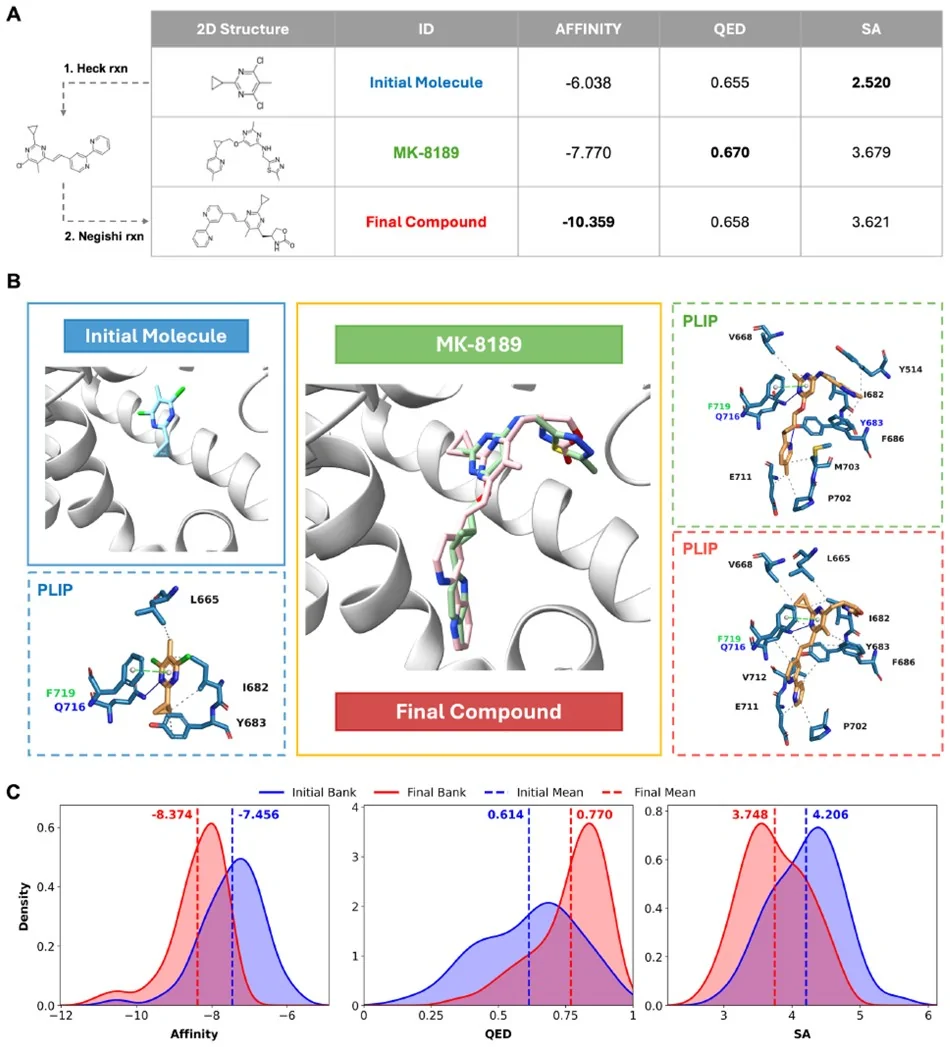
(A) Comparison of the initial molecule, MK-8189, and the best compound from the final bank in terms of 2D structures and predicted properties. (B) Binding poses and PLIP-derived interaction profiles of the three complexes. (C) Distributions of predicted binding affinity (left), QED (middle), and SA scores (right) for initial and final banks. The blue and red solid lines show the density curves of the initial bank and final bank, respectively. The dashed lines represent the average values of the properties.

Figure 2A compares the initial molecule, MK-8189, and the best final-bank compound. The MOZAIC compound was generated through two C–C coupling steps, a Heck reaction followed by a Negishi coupling. Its predicted binding affinity improved from −6.038 to −10.359 kcal/mol, exceeding that of MK-8189 in the same docking protocol (−7.770 kcal/mol). Although QED and SA were slightly affected by the increased molecular complexity, both values remained comparable to those of MK-8189.

The predicted binding pose of the MOZAIC best molecule also resembled that of MK-8189 (Fig. 2B). PLIP analysis showed that both compounds interacted with V668, I682, Y683, F686, P702, E711, Q716, and F719. In both cases, the pyrimidine core formed π-stacking with F719 and a hydrogen bond with Q716. The optimized compound occupied the binding pocket more broadly than the initial molecule, increasing interaction coverage around the PDE10A selectivity pocket (Bhardwaj and Purohit 2021), as observed in MK-8189.

Final-bank distributions showed simultaneous improvement in all three objective components (Fig. 2C). Mean binding affinity, QED, and SA shifted from −7.456 kcal/mol, 0.614, and 4.206 in the initial bank to −8.374 kcal/mol, 0.770, and 3.748 in the final bank, respectively. These results demonstrate that MOZAIC can optimize predicted binding affinity while maintaining balanced drug-like and synthetic-accessibility profiles.

#### 3.1.2 Effect of molecular diversity consideration for initial bank construction on MOZAIC result

**Figure 3:**
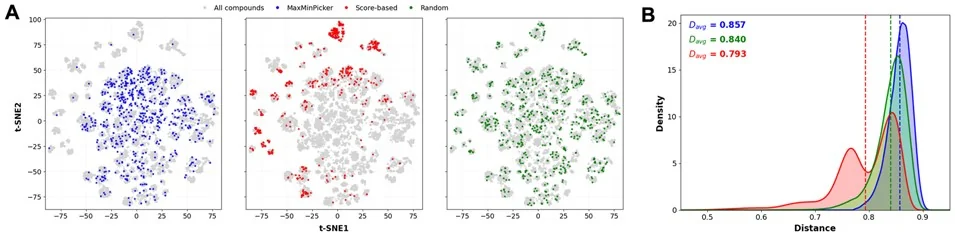
(A) A t-SNE projection of initial banks selected by three methods: MaxMinPicker (left, blue), Top-score selection (middle, red), and random selection (right, green). The generated 10 000 compounds are colored grey. (B) Pairwise distance distributions (solid line) and average distance (dashed line) of each initial bank compound.

Because CSA is population-based, its performance depends on the initial population. To assess the effect of initial-bank selection, we compared three strategies: RDKit MaxMinPicker, which prioritizes molecular diversity; score-based selection, which selects top-ranked compounds using QED + SA; and random sampling. From a pool of 10 000 generated molecules, 600 compounds were selected by each method and compared using ECFP-based molecular distances and t-SNE visualization (Fig. 3).

As anticipated, MaxMinPicker produced the most diverse initial bank. Its mean pairwise distance was 0.857, compared with 0.840 for random selection and 0.793 for score-based selection, indicating broader coverage of the generated chemical space. In the t-SNE projection, MaxMinPicker-selected compounds were also distributed more broadly across the candidate pool, whereas score-based selection was concentrated in a narrower region. These results confirm that explicitly considering molecular distance during initial-bank construction increases structural diversity before CSA optimization.

This initial diversity affected the subsequent bank update process. Supplementary Data Fig. S3, available as supplementary data at *Bioinformatics* online shows the alterations in the three-objective metrics during the optimization process. As CSA optimization proceeded, MaxMinPicker and random selection improved all three objective components, whereas score-based selection mainly improved AutoDock Vina scores and showed limited improvement in QED and SA. In the score-based case, the initial bank already contained molecules with favorable QED and SA values, leaving binding affinity as the dominant driver of later updates. In contrast, the more diverse initial banks provided structurally broader starting points, allowing CSA to identify updates that improved multiple objectives more evenly. Consistent with this, MaxMinPicker maintained the largest *D_avg_* not only in the initial bank but also in the final bank, suggesting that diversity-aware initialization helps preserve molecular diversity through optimization.

Reaction usage also depended on the initial-bank selection strategy (Supplementary Data Fig. S4, available as supplementary data at *Bioinformatics* online). MaxMinPicker and random selection used 15 reaction types in the initial banks, while score-based selection used 11. During optimization, the Stille reaction was enriched in all three methods, but the degree of enrichment differed. The Stille reaction accounted for 17.4% of reaction usage in MaxMinPicker, compared with 27.4% in random selection and 27.2% in score-based selection. Thus, although all methods exploited favorable reaction types, MaxMinPicker avoided excessive reliance on a single reaction. This broader reaction usage was reflected in the reaction-distribution SSE values of 0.880, 0.816, and 0.865 for MaxMinPicker, random selection, and score-based selection, respectively. These results indicate that diversity-based initialization promotes more balanced reaction-space exploration.

To assess reproducibility, MOZAIC was run five times with different random seeds for each selection method (Supplementary Data Fig. S5, available as supplementary data at *Bioinformatics* online). All three methods reached comparable final QED, SA, and AutoDock Vina values, indicating that diversity-aware initialization did not compromise final objective performance. However, bank diversity, the number of enriched reactions, the number of actively used reactions, and reaction-usage SSE consistently followed the order MaxMinPicker > random > score-based selection. Therefore, a diverse initial bank expands structural and reaction-space exploration while maintaining comparable optimization quality, supporting the use of MaxMinPicker for initial-bank construction.

#### 3.1.3 Influences of hyperparameters on the performance of MOZAIC

Because CSA is stochastic, we examined the effects of key hyperparameters on MOZAIC performance. First, we analyzed bank size using a molecular pool of 1000 compounds generated from the starting molecule. The bank size was varied from 1 to 500, and changes in *D_avg_*, minimum pairwise distance *D_min_*, and the number of unique Bemis–Murcko scaffolds were monitored (Supplementary Data Fig. S6, available as supplementary data at *Bioinformatics* online). *D_avg_* plateaued near a bank size of 60, while nearly every selected molecule up to this point still contributed a new scaffold. This indicates that a bank size of 60 provides a practical balance between molecular diversity and computational cost.

We next evaluated the number of CSA rounds by increasing the round number from 1 to 10 and tracking objective-function improvement (Supplementary Data Fig. S7, available as supplementary data at *Bioinformatics* online). Approximately 75% of the total improvement observed over 10 rounds was achieved within the first three rounds. Additional rounds produced only marginal gains, suggesting that three CSA rounds are sufficient for efficient optimization in the tested benchmark. Based on these results, we used a bank size of 60 and a three-round CSA protocol as the default setting.

We further assessed robustness by varying bank size and objective-function weights relative to the baseline setting of bank size 60 with equal weights (Supplementary Data Fig. S8, available as supplementary data at *Bioinformatics* online). Changing the bank size to 30, 90, and 120 mainly affected the number of actively used reactions and runtime, whereas average final-bank quality remained largely unchanged. In particular, smaller banks reduced runtime but explored fewer reactions, while larger banks increased reaction usage at higher computational cost. When each objective weight was reduced from 1 to 0.5 while the other two were fixed, the corresponding objective was primarily degraded, with limited effects on the other metrics. These results indicate that MOZAIC is robust to moderate hyperparameter changes, while bank size mainly controls the trade-off between exploration breadth and runtime.

### 3.2 Optimizing a compound targeting TrmD and comparing it to CReM-dock and REINVENT4

In order to assess the performance of MOZAIC and to compare it with other molecule optimization methods, TrmD was selected as the next benchmark. CReM-dock and REINVENT4 were selected for comparison. Similar to MOZAIC, CReM-dock employs AutoDock Vina and a genetic algorithm to append fragments, with the objective of optimizing a query molecule. Conversely, REINVENT4 utilizes reinforcement learning. Indole-5-carboxamide, the starting molecule, with an IC_50_ of 160 μM against the target protein, was selected. Wilkinson and colleagues (Wilkinson *et al*. 2023) optimized the molecule to BDBM50613282, with an IC_50_ of 1.3 nM.

Table 1 shows the top three molecules and their properties obtained by the three programs. Comparing the top three molecules obtained by MOZAIC and CReM-dock, MOZAIC-generated molecules exhibited stronger predicted binding affinities with a mean of −9.683 kcal/mol compared to the initial molecule (−6.891 kcal/mol), and even lower than the optimized molecule (−8.220 kcal/mol). Conversely, the SA score and QED demonstrate a similarity to, or in some cases, a lower performance than that of the optimized compound. CReM-dock also successfully produced the molecules exhibiting better binding affinities compared to the initial compound and the optimized one, with an average binding energy of −8.428 kcal/mol.

**Table 1:** Comparison of the top three compounds from MOZAIC, CReM-dock, and REINVENT4 by two-dimensional structures, AutoDock Vina scores, QED, SA scores, ligand efficiency, and Tanimoto similarity to the initial molecule.

	2D Structure	AutoDock Vina Score (kcal/mol)	QED	SA	Ligand efficiency	Similarity to the initial molecule
Initial Molecule	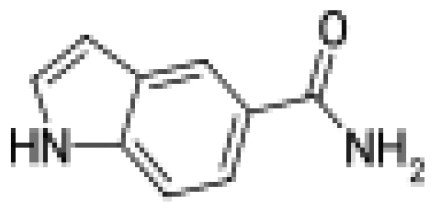	−6.891	0.648	1.969	0.574	1
Optimized Molecule (Wilkinson et al.)	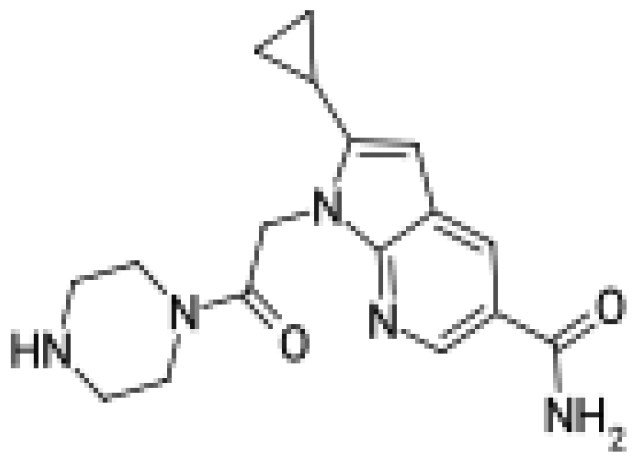	−8.220	0.852	2.684	0.343	0.141
MOZAIC	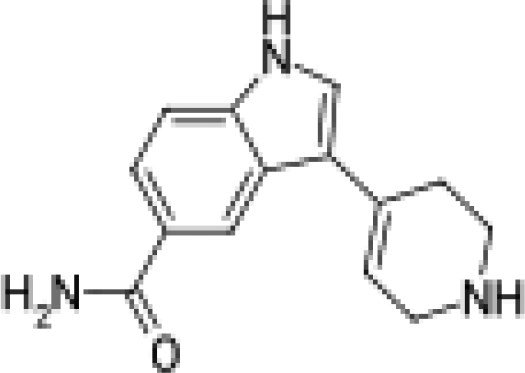	−8.597	0.748	2.571	0.478	0.253
	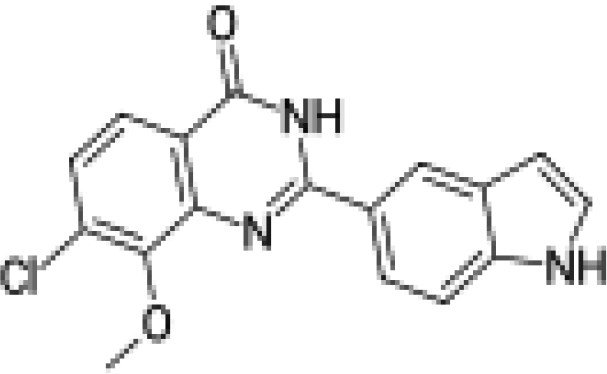	−9.359	0.590	2.497	0.407	0.226
	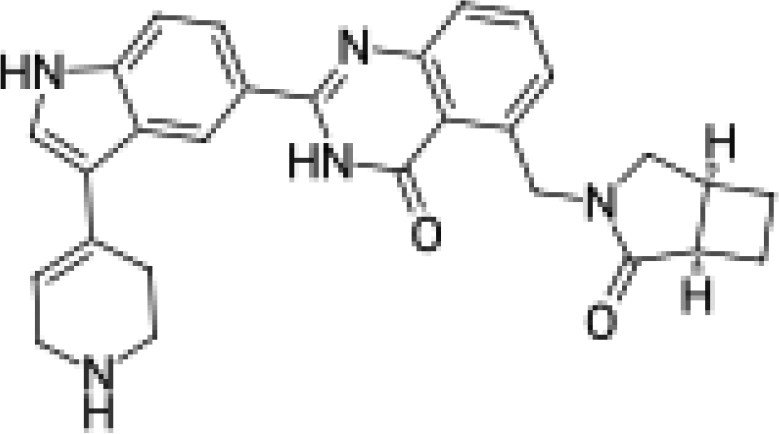	−11.094	0.427	3.853	0.317	0.078
CReM-dock	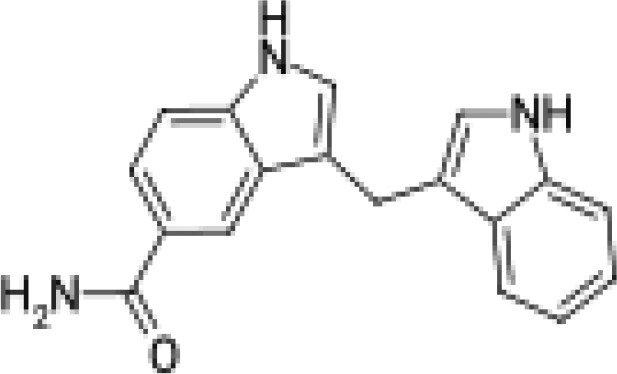	−8.617	0.531	2.128	0.392	0.238
	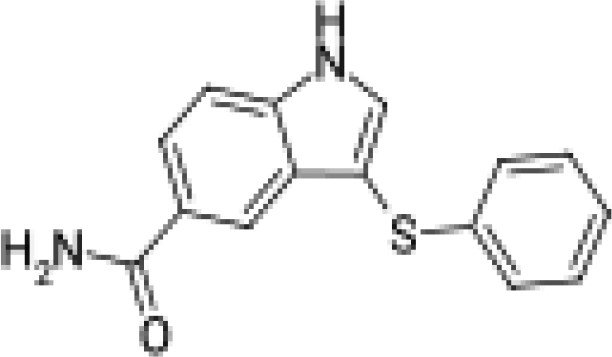	−7.618	0.764	2.120	0.401	0.247
	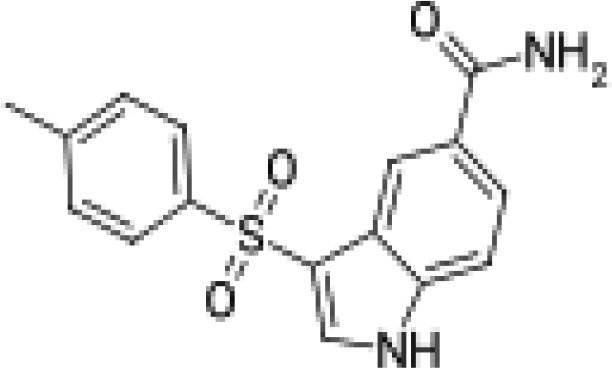	−9.048	0.777	2.095	0.411	0.235
REINVENT4	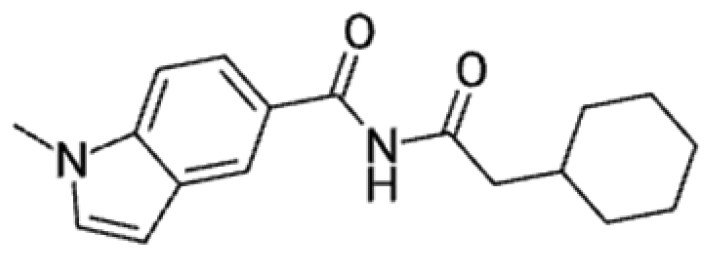	−8.501	0.944	2.175	0.386	0.113
	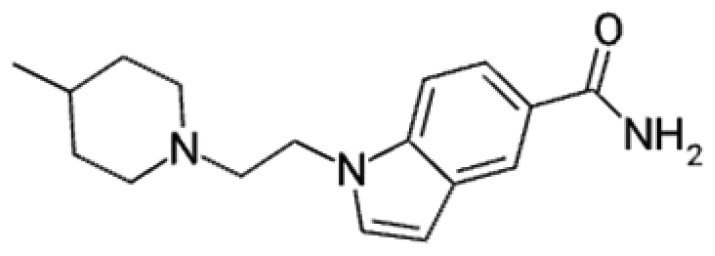	−8.443	0.938	2.145	0.402	0.195
	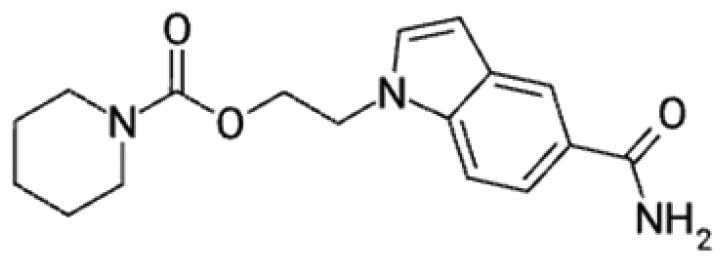	−8.413	0.941	2.185	0.366	0.181

Ligand efficiency is defined as the absolute Vina score divided by the number of heavy atoms. Tanimoto similarity is computed from ECFP fingerprints (radius 4, 2048 bits).

A comparison of the top three molecules from each method reveals that MOZAIC produced stronger and larger binders than CReM-dock. The AutoDock Vina scoring function uses an atom-pairwise additive scheme, which might favor larger molecules by assigning them higher docking scores (Xu *et al*. 2022). To remove the size-related bias, ligand efficiency, defined as the absolute docking score divided by the number of heavy atoms, was calculated (Reynolds *et al*. 2008). The arithmetic mean of the top three molecules from each program yielded the same value: 0.401. On the other hand, CReM-dock showed better QED and SA values than MOZAIC. The average values of CReM-dock are 0.691 and 2.114, while those of MOZAIC are 0.588 and 2.974 for QED and SA, respectively. However, the top three CReM-dock molecules show minimal variation—primarily small substituents added to the same positions—resulting in limited molecular diversity. In contrast, MOZAIC generates structurally diverse compounds, with examples that include ring formation and distinct modifications. Consistent with this observation, the final 60 compounds of MOZAIC exhibit greater divergence from the initial molecule. The average Tanimoto similarity to the initial molecule is 0.135, ranging from 0.074 to 0.253, whereas the top 60 CReM-dock compounds remain closer to the initial structure, from 0.198 to 0.412, with an average of 0.285. This increased deviation is accompanied by broader chemotype coverage. To evaluate this, we analyzed the BMS of the top 60 compounds from both programs. MOZAIC generated more unique scaffolds than CReM-dock (45 vs. 32). The SSE of the scaffolds was higher for MOZAIC than for CReM-dock (0.943 vs. 0.805), indicating a more even distribution across scaffold classes. Moreover, the most frequent scaffold accounted for a smaller proportion of compounds in MOZAIC than in CReM-dock (0.133 vs. 0.367). These findings suggest that MOZAIC explores a broader and more balanced scaffold space. In addition, CReM-dock expands fragments by attaching new moieties to available hydrogen atoms, with spatial and size restrictions. In contrast, MOZAIC performs reaction-based modifications guided by SMARTS rules, where the reaction site is user-specified, with no explicit geometric constraint. This allows MOZAIC to offer a route-level interpretability to synthesize molecules, alongside broader chemical novelty.

Additionally, MOZAIC was compared to REINVENT4 using R-group replacement mode. As in MOZAIC, the indole-carboxamide and the two functional-group atoms were used as attachment points; execution details are provided in Supplementary Data, available as supplementary data at *Bioinformatics* online. From 100 000 generated molecules, the top 60 were docked and reranked using the same QED, SA, and Vina objective and docking protocol as MOZAIC. REINVENT4 showed more favorable QED and SA among the top three compounds than MOZAIC (mean QED 0.941 vs. 0.588 and SA 2.168 vs. 2.974), whereas MOZAIC produced stronger predicted binders with higher ligand efficiency (mean Vina −9.683 vs. −8.452 kcal/mol and ligand efficiency 0.401 vs. 0.385). Both methods generated compounds substantially different from the initial molecule, with comparable average Tanimoto similarities of 0.135 and 0.126 for MOZAIC and REINVENT4, respectively. MOZAIC produced slightly more unique BMS than REINVENT4 (45 vs. 42), whereas REINVENT4 showed a more even scaffold distribution, with a higher SSE of 0.969 (MOZAIC: 0.943). These differences reflect their objective functions: REINVENT4 optimized QED and SA directly, while Vina was applied only post hoc, unlike MOZAIC. Overall, the two methods showed complementary strengths, while MOZAIC provided synthetically traceable, docking-guided optimization and reaction site that could guide generative models such as REINVENT4.

### 3.3 Optimizing compounds in the absence of relevant receptor structures

Because MOZAIC uses AutoDock Vina, its performance may depend on receptor conformation. To evaluate this sensitivity, MOZAIC was applied to ADRB2 using both the carazolol-bound crystal structure, whose ligand was dissimilar to the starting molecule, and an AF3-predicted structure. The optimized compounds were compared with the top five pK_i_ ligands from the same ChEMBL cluster as the starting molecule (Table 2).

**Table 2:** Comparison of the final compounds optimized by MOZAIC with predefined reference ligands on the ADRB2 crystal structure (PDB ID: 2RH1) and AF3 structure.

	2D Structure	Vina score (2RH1)	Vina score (AF3)	QED	SA	Similarity to the starting molecule	pK_i_
Starting fragment	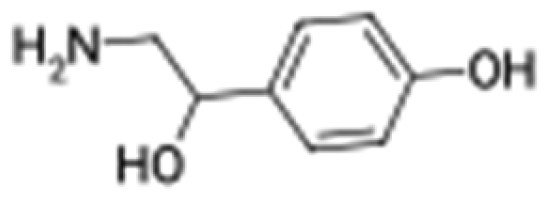	−6.801	−6.369	0.575	2.406	1	
MOZAIC (2RH1)	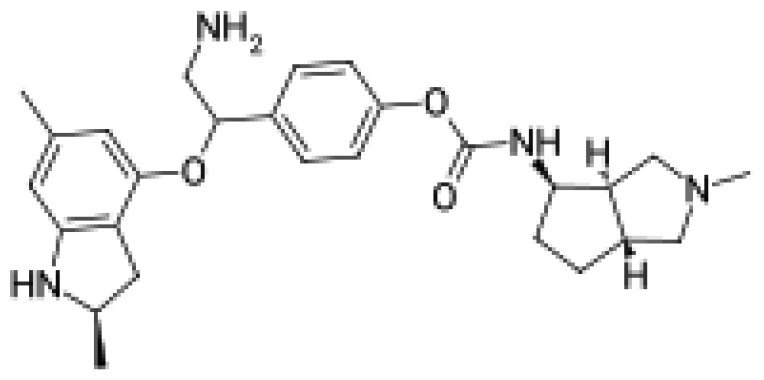	−10.894		0.602	4.254	0.098	
MOZAIC (AF3)	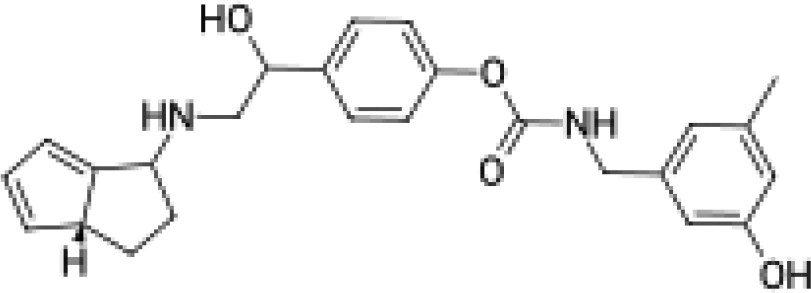		−11.198	0.546	3.834	0.127	
Reference Compounds	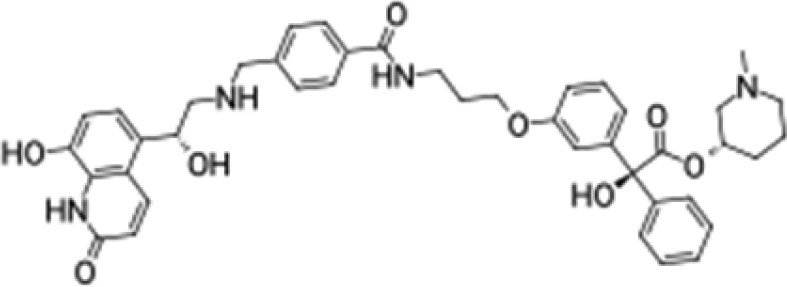	−10.203 ± 1.190	−8.382 ± 1.602	0.068	4.047	0.051	10.6
	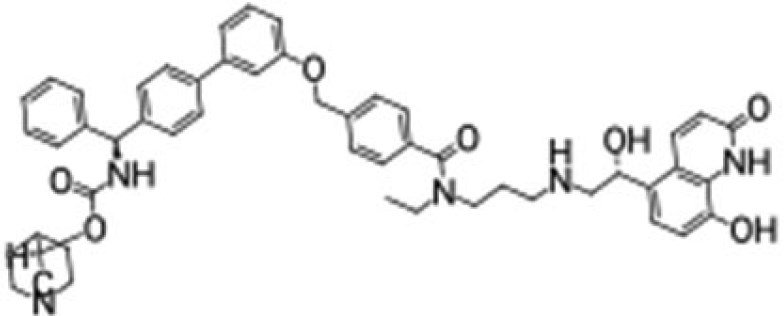	−8.839 ± 2.192	−5.921 ± 2.214	0.058	5.011	0.065	10.3
	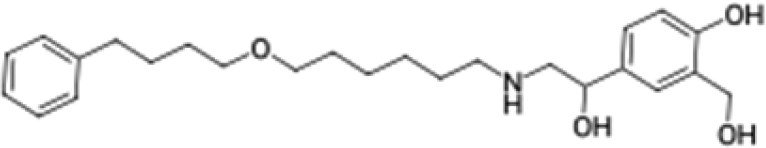	−8.501 ± 0.193	−8.727 ± 0.296	0.310	2.720	0.108	9.7
	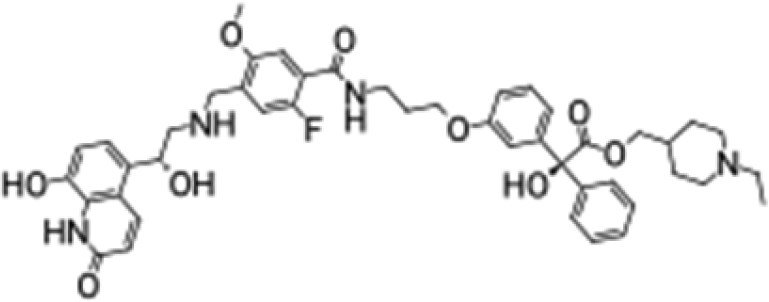	−10.018 ± 1.779	−5.057 ± 1.895	0.052	4.099	0.048	9.3
	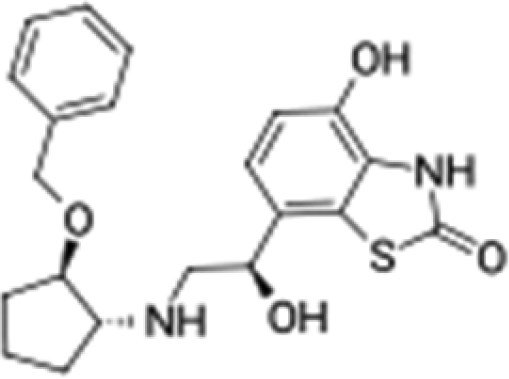	−10.227 ± 0.120	−10.383 ± 0.085	0.489	3.539	0.091	9.3

Vina scores and their standard deviations of reference ligands were obtained by 5 runs.

The best MOZAIC compounds showed Vina scores comparable to those of reference ligands with pK_i_ > 9, while maintaining more favorable QED and comparable SA scores. They also showed low similarity to the starting fragment, comparable to the reference ligands, indicating that MOZAIC generated structurally distinct optimized compounds under receptor-structure variation.

MOZAIC initially identified three reactive sites in the starting molecule: hydroxyl, primary amine, and phenol groups. Although most initial-bank compounds substituted all three sites (45/60 molecules for both structures), the final banks were dominated by bi-site reacted molecules, with 43 of 60 compounds for both receptor structures. These compounds balanced affinity and molecular properties, with mean values of −9.391 kcal/mol, 0.672, and 3.692 for Vina score, QED, and SA, respectively, when optimized using X-ray structure. Uni-site products showed favorable QED (0.798) and SA (3.247) but weaker affinity (−8.437 kcal/mol), whereas tri-site substitution improved affinity (−9.709 kcal/mol) at the cost of drug-likeness (0.650) and synthesizability (4.188).

Similar to the X-ray structure, the AF3 structure-based optimized molecule showed a comparable binding affinity and improved QED and SA scores compared with the reference molecules. The reference molecules showed relatively weaker binding affinities when docked to the AF3-predicted structure. This suggests MOZAIC is capable of identifying diverse chemotypes that retain docking performance even under receptor-structure uncertainty.

### 3.4 Customization of physicochemical objective functions in MOZAIC

Because the MOZAIC objective function is modular and implemented in Python 3, its components can be replaced to optimize alternative molecular properties. To test this flexibility, QED and SA were replaced with ESOL-predicted logS and AutoDock Vina score to improve solubility while maintaining binding affinity for PPARγ ligands. CHEMBL375270 was selected as the starting compound, and the top three MOZAIC-optimized molecules are shown in Table 3.

**Table 3:** The starting compound selected from ChEMBL that binds to PPARγ and its top three optimized compounds by MOZAIC.

Compound	Structure	AutoDock Vina (kcal/mol)	Predicted logS
CHEMBL375270	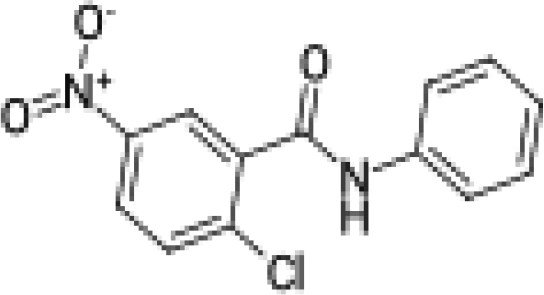	−7.666	−4.424
MOZAIC	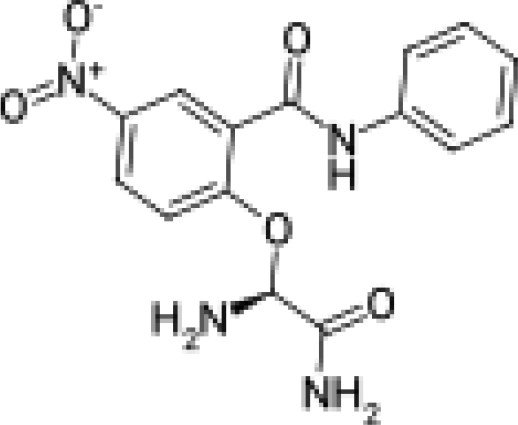	−8.272	−2.854
	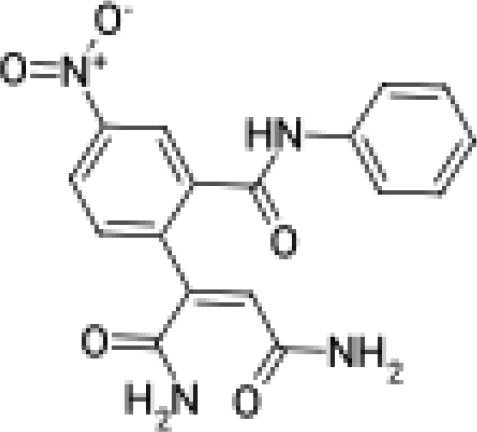	−8.636	−3.149
	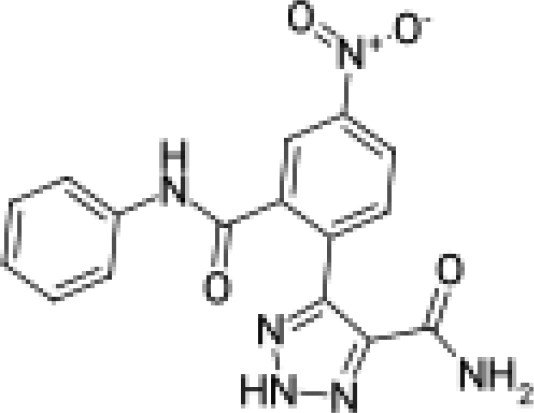	−8.796	−3.614

MOZAIC identified two reactive sites in the starting molecule, amine/amide and halide. Although the initial bank was dominated by bi-site-reacted compounds (59/60), the final bank mainly contained uni-site-substituted molecules, with only two bi-site-reacted compounds among 60. This shift likely reflects the Y- or T-shaped PPARγ binding pocket (Kroker and Bruning 2015), where dual-site expansion could introduce steric clashes and excessive hydrophobicity. Consistent with this, the initial bank compounds exhibited poor solubility, with a mean logS of −8.563 (±1.278), reflecting the predominance of bi-site-reacted compounds containing two hydrophobic fragments. The inconsistent fit of these compounds within the binding pocket also resulted in a high Vina score variance (−7.797 ± 2.628), whereas the final solutions achieved a stable and favorable Vina score profile (−8.599 ± 0.561, range: −10.540 to −7.612) with significantly improved solubility (−4.647 ± 0.593).

The top three optimized molecules replaced the chlorine atom para to the nitro group using Halide_and_Alcohol or Stille reactions. Compared with the starting molecule, both predicted affinity and solubility improved, with mean Vina score and logS values of −8.568 kcal/mol and −3.206, respectively. The substitutions increased molecular polarity while improving the predicted binding affinity (Supplementary Data Fig. S9, available as supplementary data at *Bioinformatics* online). Notably, while the nitro-group position coincides with that in the initial structure, the three optimized compounds adopt altered but mutually consistent binding modes, in which the para substituent is redirected into the interior subpocket. This reorientation appears to minimize steric hindrance while increasing volumetric occupancy and shape complementarity within the confined Y-shaped cavity, leading to tighter packing in the buried region and a common pose family across the optimized derivatives.

We further tested objective-function flexibility by replacing QED and SA with logS and SCScore in the PDE10A benchmark. SCScore is a synthetic-feasibility metric trained on 12 million Reaxys reactions to estimate the expected number of reaction steps required for synthesis (Coley *et al*. 2018). MOZAIC improved all three alternative objectives over the run (Supplementary Data Fig. S10, available as supplementary data at *Bioinformatics* online): Vina score from −7.64 to −7.87 kcal/mol, logS from −5.10 to −3.22, and SCScore from 4.26 to 3.71. These results show that the modular objective can be retargeted without algorithmic modification. Post hoc MM-GBSA rescoring correlated only weakly with Vina score (Pearson *r* = 0.20) and reordered candidate rankings (Spearman *ρ* = 0.50), supporting its use as a complementary prioritization filter. Consequently, the program successfully optimized the initial molecule to identify solution candidates, achieving a favorable balance between enhanced solubility and binding potency.

## 4 Conclusion

In this study, we presented MOZAIC, a structure-based fragment-growing framework that integrates reaction rules with the CSA algorithm. A powerful global optimization algorithm, CSA, enables MOZAIC to rigorously explore the chemical space defined by synthetic rules, ensuring both optimization quality and structural diversity. The application of this framework to benchmarks demonstrated its advantages. First, MOZAIC could suggest potent compounds comparable to the lead compound in terms of the docking score. Second, facilitated by CSA, MOZAIC produced compounds with higher scaffold SSE and lower structural redundancy, effectively avoiding the generation of repetitive analogues. Third, the program identified alternative chemotypes that retained favorable docking scores, demonstrating robustness against receptor-structure uncertainty. Finally, it showed flexibility in choosing physicochemical properties to be optimized.

However, several aspects require improvement. The current implementation relies on AutoDock Vina, which has limited accuracy in absolute quantitative affinities, as it does not adequately account for target flexibility or solvent effects (Pantsar and Poso 2018). Additionally, the chemical space is explored via the predefined reaction rules, restricting molecular diversity (Saldívar-González *et al*. 2020). To address these limitations, the future work will focus on expanding the accessible chemical space by integrating a broader set of SMARTS-based reactions. Advanced scoring methods, such as quantum mechanics-based potentials or Vina-GPU (Tang *et al*. 2024), will be incorporated into the scoring function to enhance accuracy and speed. In addition, recently developed co-folding models such as Boltz-2 (Passaro *et al.* 2025) could provide an external assessment by jointly predicting binding conformations and affinities. For blind docking scenarios, tools such as Fpocket (Le Guilloux *et al*. 2009) and P2Rank (Krivák and Hoksza 2018) could be incorporated for binding site prediction.

## Author contributions

Jinhyeok Yoo (Formal analysis [lead], Data Curation [lead], Software [lead], Investigation [lead], Visualization [lead], Writing–original draft [lead]), and Woong-Hee Shin (Conceptualization [lead], Project administration [lead], Supervision [lead], Writing–original draft [supporting], Writing–review & editing [lead])

## Supplementary Material

btag595_Supplementary_Data

## Data Availability

All benchmarked data are available at https://github.com/kucm-lsbi/MOZAIC/tree/main/benchmark_results/.

## Code availability

The source code for MOZAIC is available at https://github.com/kucm-lsbi/MOZAIC/.
