# Supplementary material for: MOZAIC: compound growth via in silico reactions and global optimization using Conformational Space Annealing
Source: Bioinformatics. 2026 Aug 7;42(8):btag595. doi: 10.1093/bioinformatics/btag595 (PMC13493189; doi:10.1093/bioinformatics/btag595)
Supplement: Supplementary file 1 [file btag595_supplementary_data.pdf]

Supplementary Data for

MOZAIC: Compound Growth via *In Silico* Reactions and Global Optimization using Conformational Space Annealing

*Jinhyeok Yoo and Woong-Hee Shin**

Department of Biomedical Informatics, Korea University College of Medicine, Seoul, 02708, Republic of Korea

Correspondence to:

Woong-Hee Shin

E-mail: whshin@korea.ac.kr

Phone: +82-2-3407-2185

Fragment Library Construction Process

1. We downloaded 'Annotated' molecules from ZINC20 and applied initial filters of logP < 3 and molecular weight < 300, resulting in ~450 milion SMILES entries.

2. Physicochemical properties of entries were calculated using RDKit, including the number of hydrogen bond donors/acceptors and rotatable bonds, and further filtered according to the Rule of Three (Ro3) (Jhoti et al. 2013) for fragments.

3. Redundant entries were removed using canonical SMILES, duplicates were collapsed into single representatives, leaving approximately 80 million unique fragments.

4. Finally, fragments containing functional groups required for AutoClickChem, Robust and ChemoDOTS reactions were selected, and the final library was constructed with up to 10,000 fragments, ordered by increasing molecular weight.

Figure S1. Process of constructing the fragment library

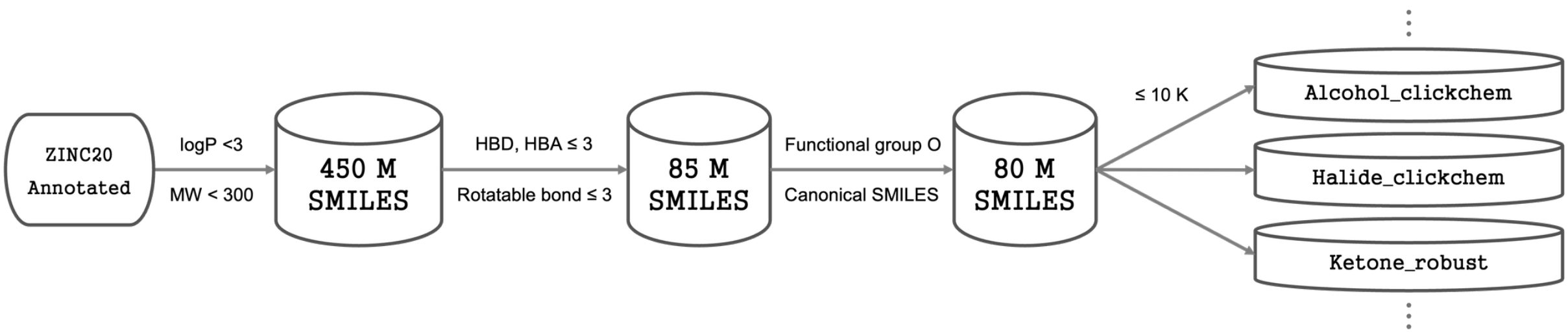

Selecting the starting fragment from ChEMBL for ADRB2

1. Collect active compounds with pChEMBL ≥ 7; 391 compounds are retained (for duplicate SMILES, only the entry with the highest pChEMBL is kept).

2. Calculate ECFP fingerprint with radius of 4, 2048 bits.

3. Clustering ligands using Butina algorithm with a Tanimoto coefficient of 0.7.

4. Derive an MCS from the top-5 pChEMBL ligands in each cluster, and use the MCS from the largest selected cluster as the starting fragment.

Figure S2. Workflow for starting fragment extraction.

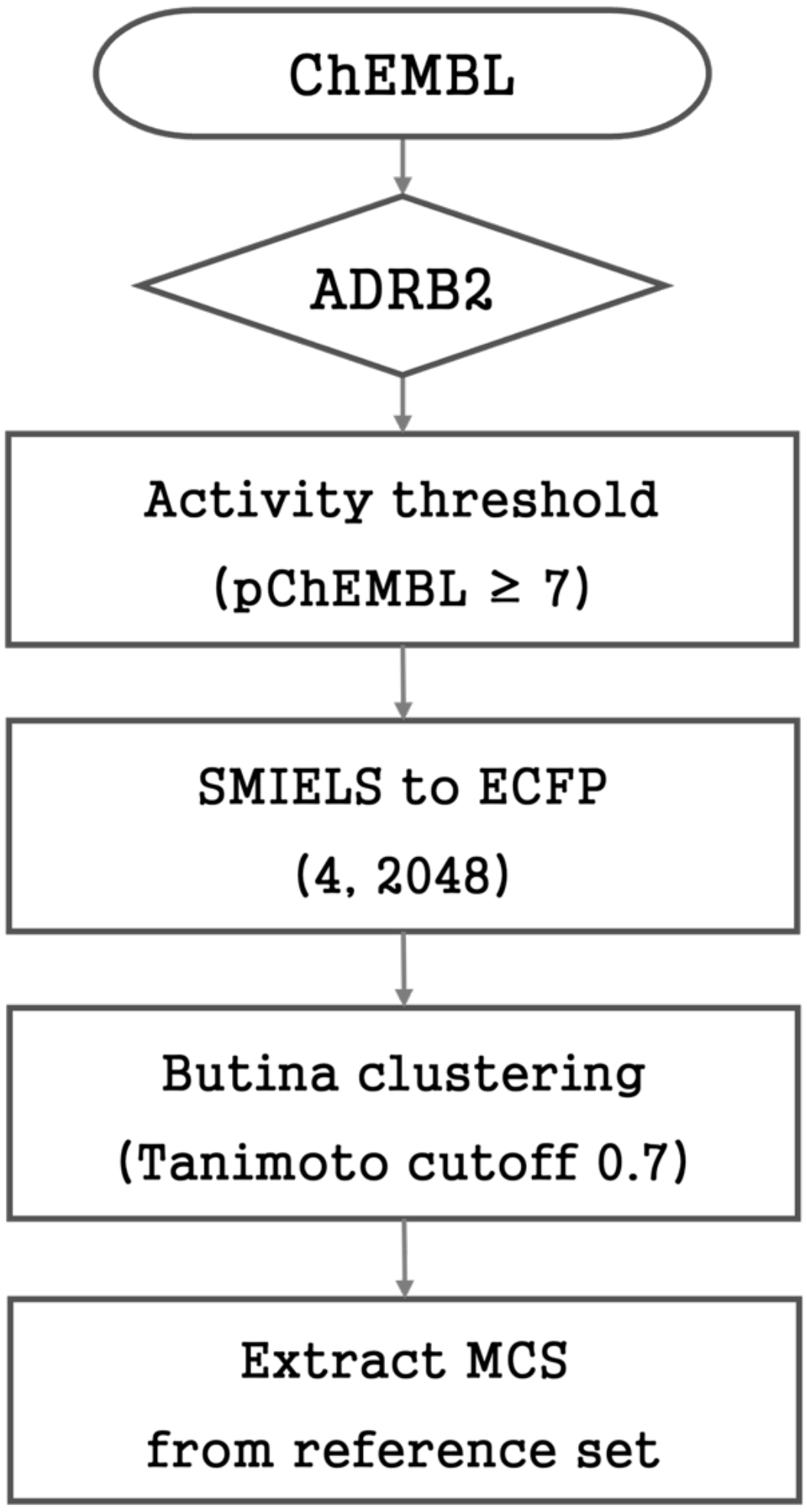

Figure S3. Tracking bank update history for the initial bank construction method: (**A**) MaxMinPicker, (**B**) random, and (**C**) score-based. Within each row, from the top, the four panels show a run-wide rescaled trajectory of the bank-mean value for each objective, per-update improvement in each objective terms, dominant improving objective with the largest improvement at each update step, and bank diversity quantified by D_{avg}. Vertical dashed lines mark CSA rounds.

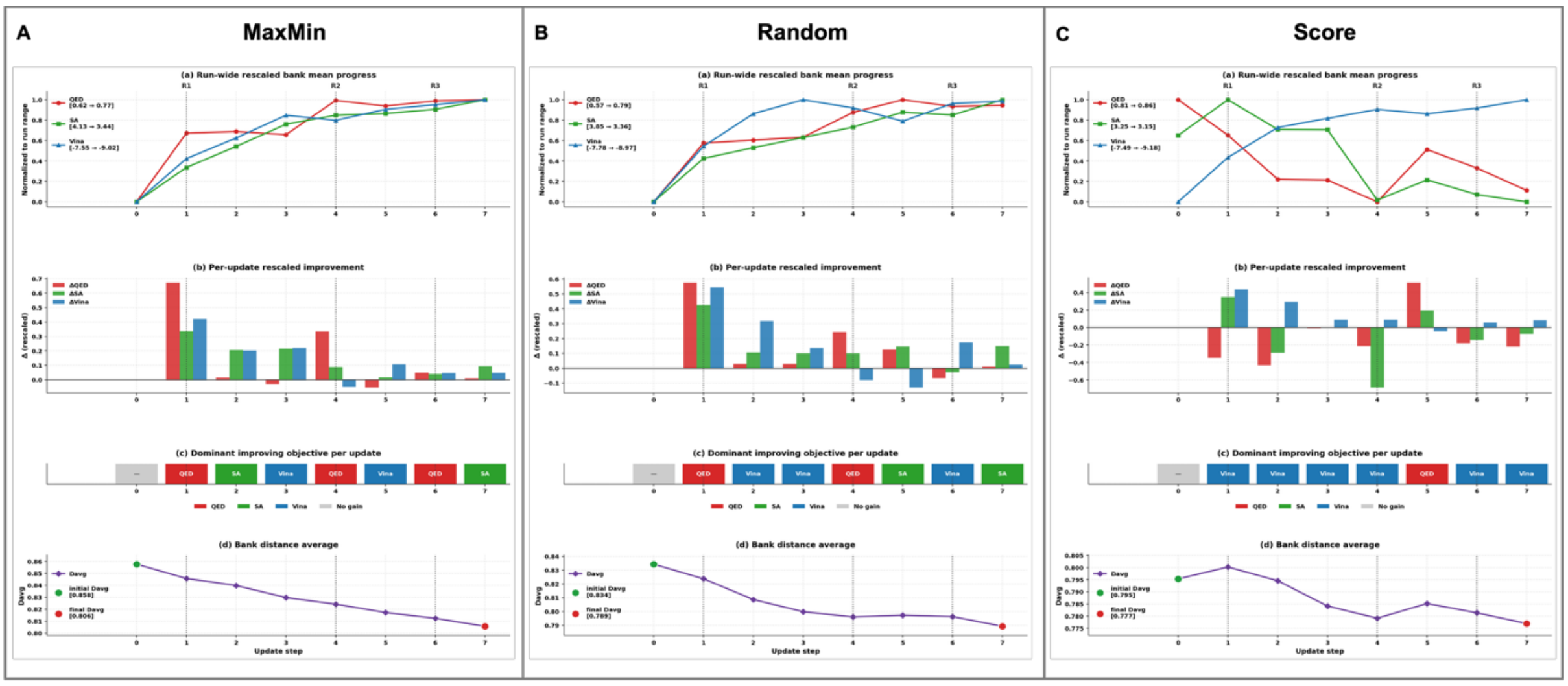

Figure S4. Tracking data of reactions used at each bank update and their enrichments for the initial bank construction method: (**A**) MaxMinPicker, (**B**) random selection, and (**C**) score-based. From the top, three panels show a stacked area plot of reaction shares across update steps, a change in reaction share over the run (Δshare = final − initial), and (c) top 10% enrichment ratio per reaction, annotated with Δshare and occurrence count (n). The enrichment ratio quantifies over- or under-representation of each reaction among the top-scoring 10% of final bank molecules.

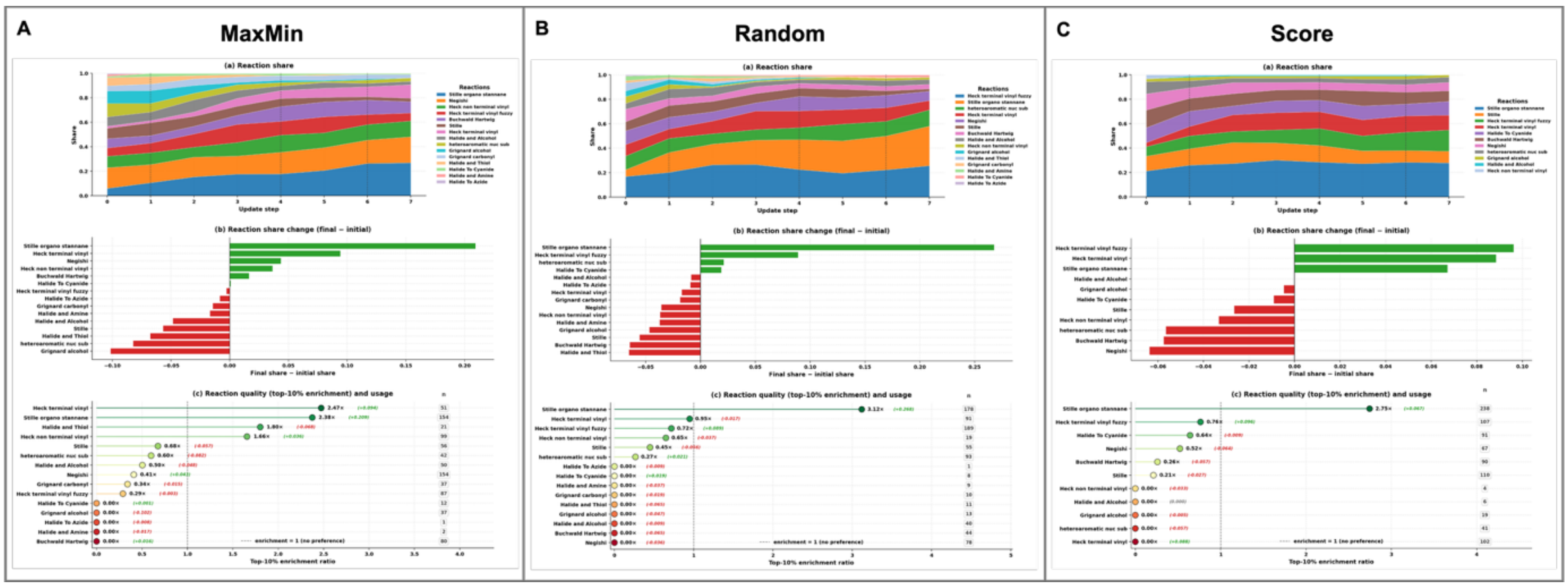

Figure S5. Summary statistics across five seeds for three initial-bank selection methods. All values are reported as mean ± SD across five independent seeds per method. (**A**) Initial and final bank-mean QED, SA, Vina score (kcal/mol), and bank diversity. (**B**) Δshare (final − initial) summed across reactions grouped by final-bank enrichment status: enriched (>1×) or non-enriched (≤1×); number of enriched reactions, defined by final-bank enrichment ratio >1×; number of actively used reactions, defined by n ≥ 10; and SSE of reaction usage, where 1 indicates a uniform distribution across reactions and values approaching 0 indicate concentration on a single reaction.

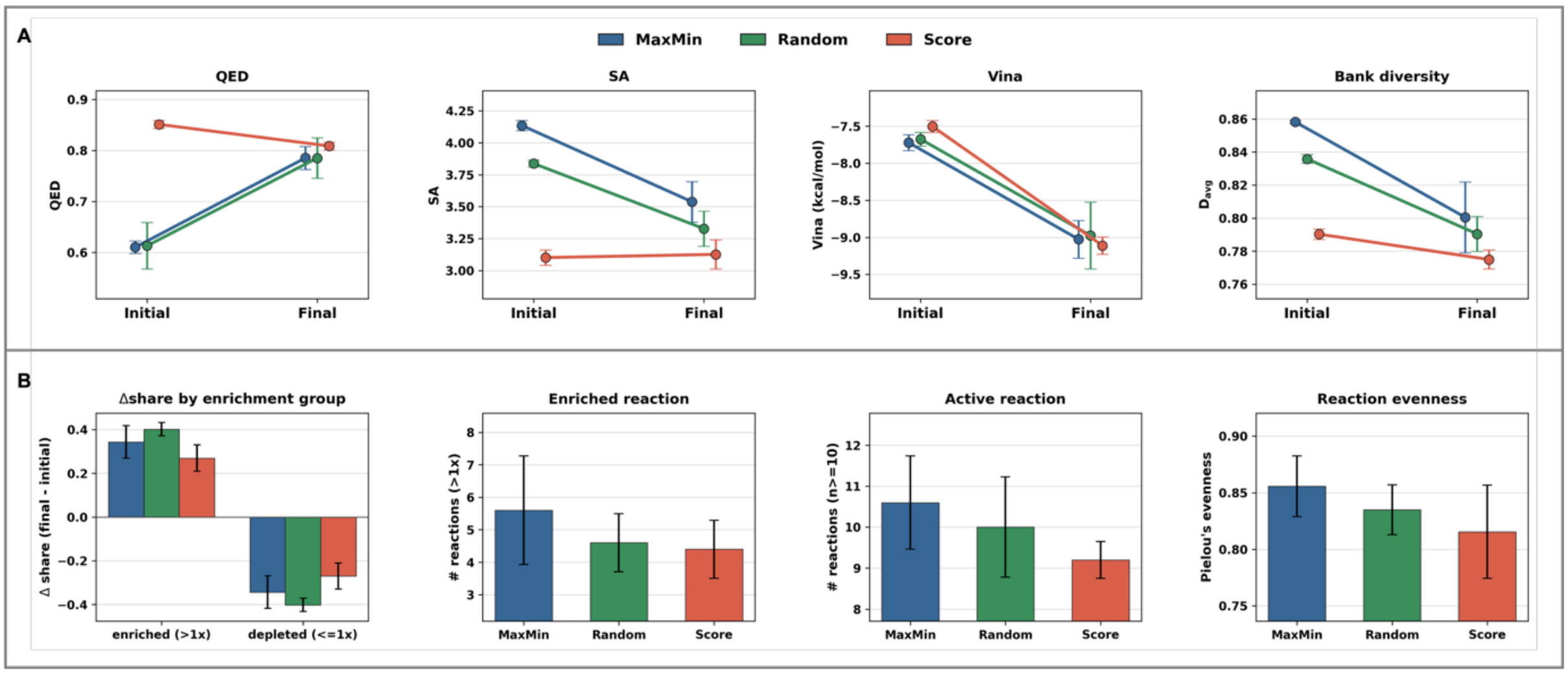

Figure S6. Diversity and scaffold coverage versus the number of molecules selected by MaxMinPicker. (**A**) Mean pairwise distance (D_{avg}, purple) and minimum pairwise distance (D_{min}, orange) among the selected molecules. The dotted vertical line marks the D_{avg} elbow (n ≈ 7), and the dashed vertical line marks the bank size used in the main analysis (n_{bank} = 60). (**B**) Number of unique Bemis–Murcko scaffolds among the selected molecules. The dotted line indicates the upper bound of one new scaffold per selected molecule.

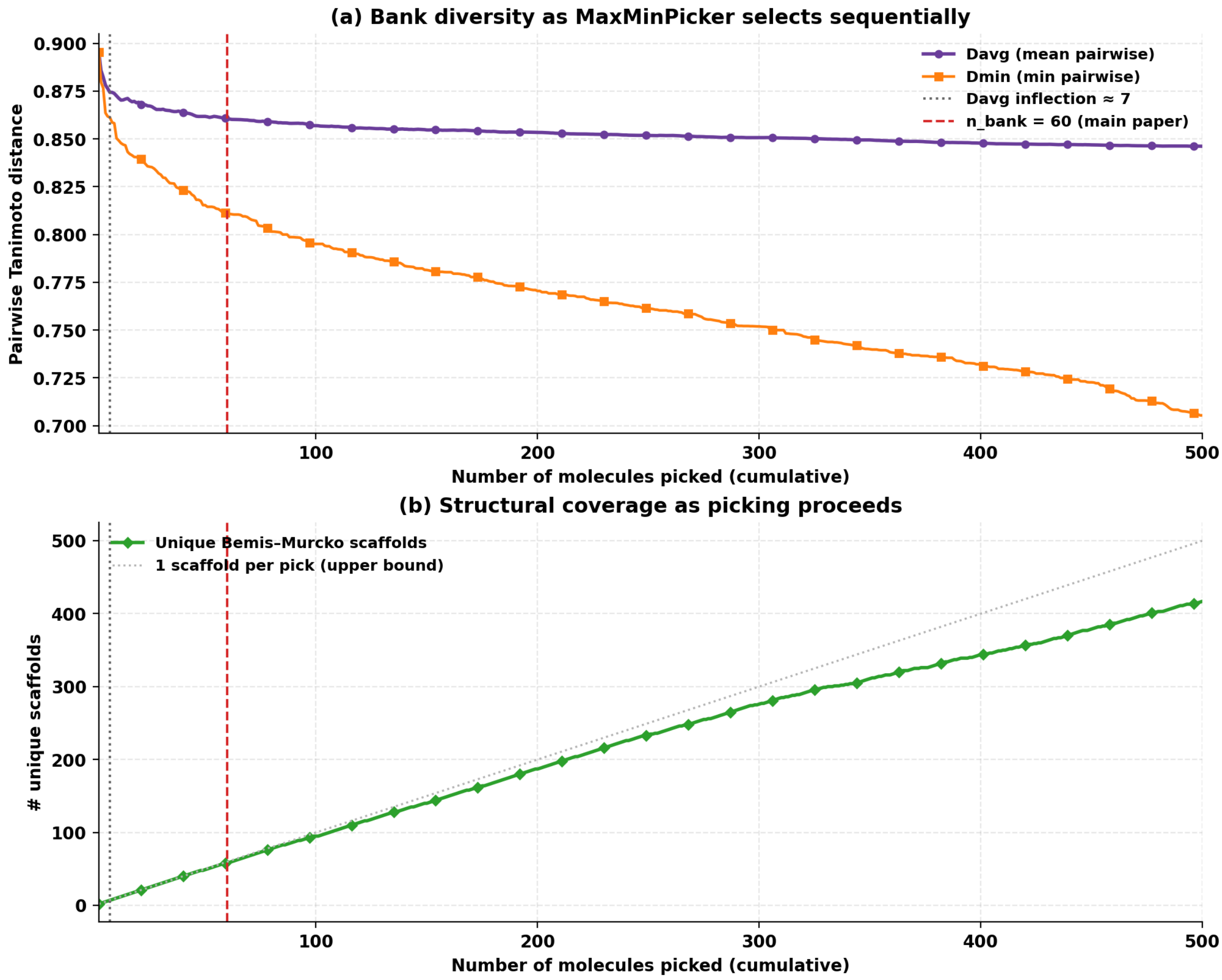

Figure S7. Round-wise optimization progress over 10 CSA rounds, averaged across five seeds. Five metrics are tracked: mean Vina, mean QED, mean SA, the normalized sum of the three objectives, and the 3D hypervolume. Each metric is direction-adjusted and rescaled per seed so that round 0 (R0) = 0 and round 10 (R10) = 1; shaded bands indicate ± SD across seeds. (Top) Cumulative normalized progress. The dotted line marks 75% of the total improvement, and the dashed vertical line marks the consensus plateau onset at R3. (Bottom) Per-round change in normalized progress, |Δ normalized progress|; the dashed horizontal line marks ε = 0.10, the threshold below which a round contributes less than 10% of the total R0-to-R10 improvement.

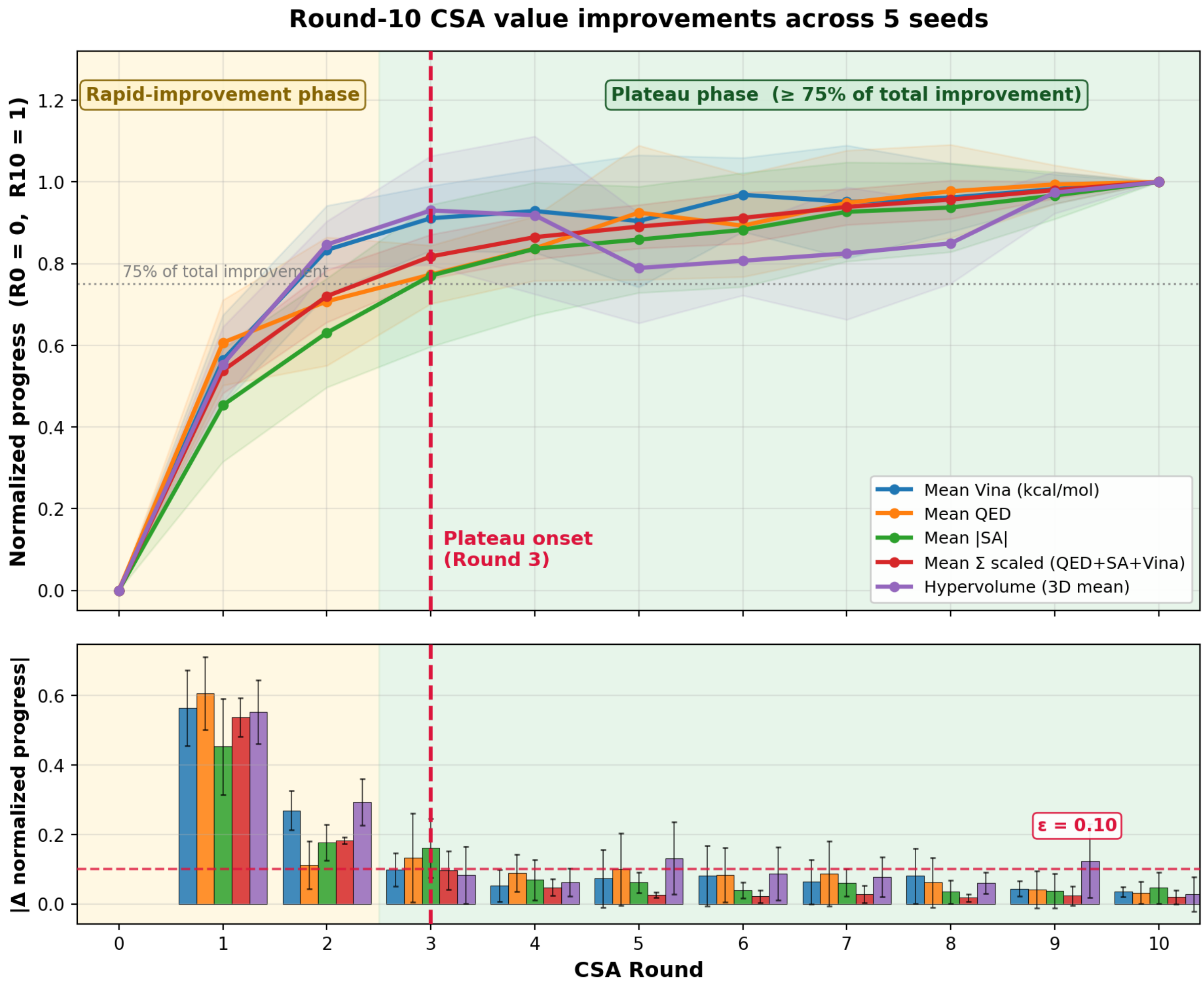

Figure S8. Sensitivity of MOZAIC outcomes to bank size (A) and objective weighting (B). Each point shows the mean percentage difference from the baseline across five seeds. The baseline is defined as bank size 60 with equal objective weights of 1:1:1. Metrics are grouped into final-bank quality (QED, −SA, −Vina, and D_{avg}); reaction repertoire, including actively used reactions (n ≥ 10) and Pielou's evenness; and computational cost, measured by total runtime. Whiskers represent the combined seed-to-seed spread of the baseline and the perturbed configuration. (A) Bank sizes of 30, 90, and 120 compared with the baseline bank size of 60. (B) Each objective weight individually set to 0.5 while the other two are held at 1, compared with the equal-weight baseline.

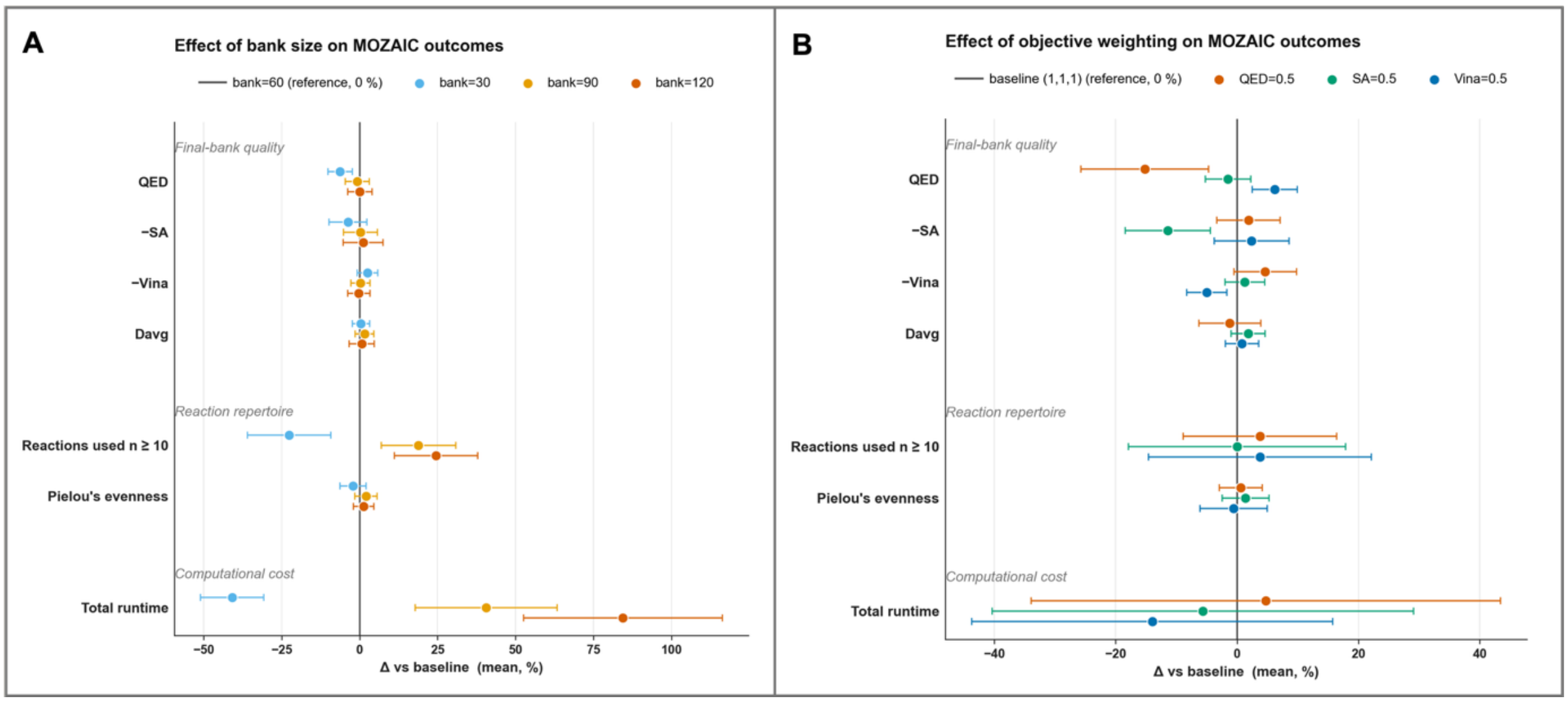

Figure S9. Superposed docking poses of the initial compound (sky blue) and MOZAIC derivatives (pink shades), illustrating pose convergence among optimized compounds.

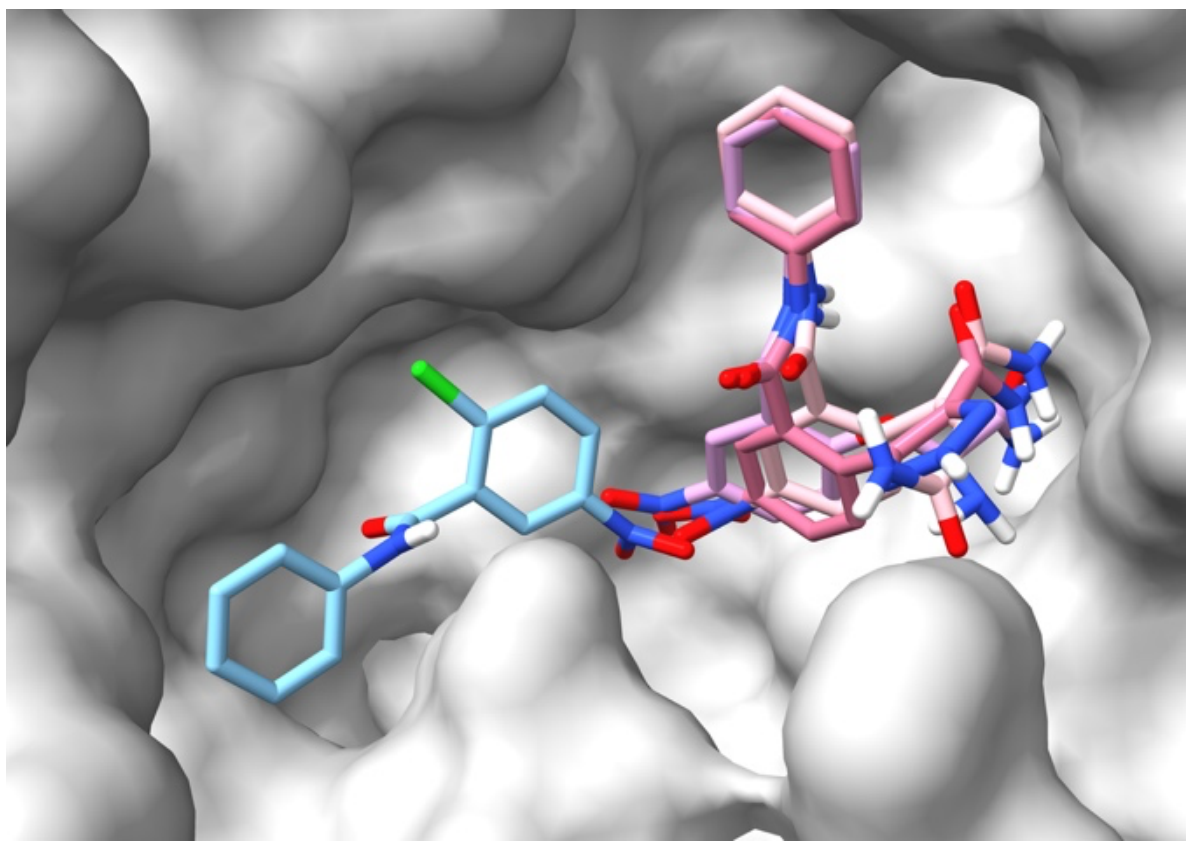

Figure S10. Alternative objective set and post hoc MM-GBSA rescoring. (**A**) MOZAIC optimization with an alternative objective set consisting of Vina, logS, and SCScore, comparing the initial and final banks. Values are mean ± SD over five independent seeds. (**B**) MM-GBSA binding free-energy estimate versus Vina score for 60 compounds from one final bank. More negative values indicate stronger predicted binding on both axes.

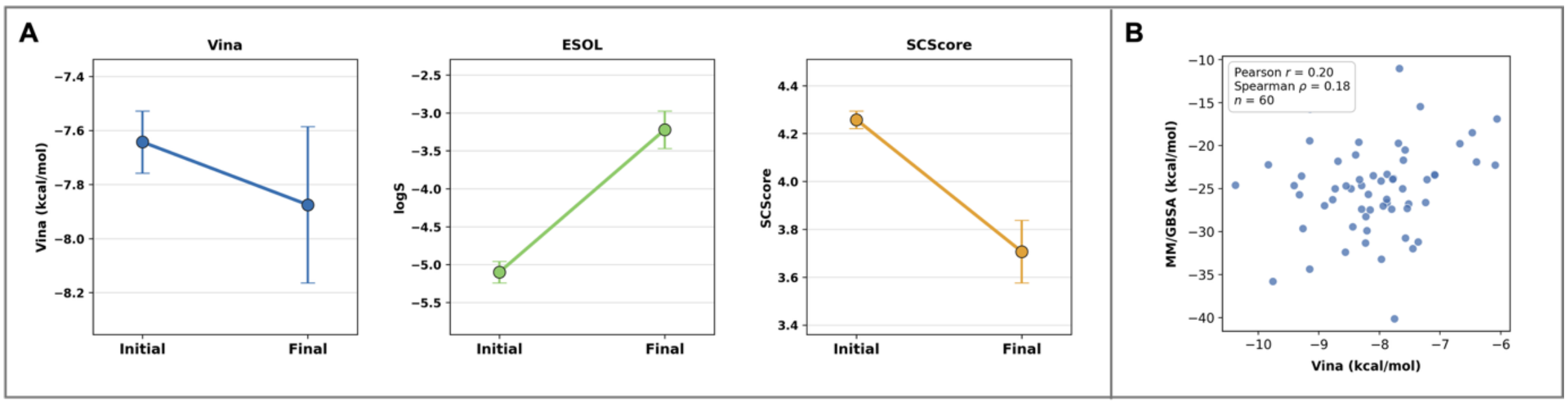

Execution of REINVENT4 R-group replacement

R-group replacement was performed using LibInvent in REINVENT4 to generate analogues of the indole-carboxamide used in Section 3.2. Since R-group replacement requires explicit specification of attachment points, three input scaffolds were prepared based on the same two functional-group atoms identified by MOZAIC: one with the amide nitrogen designated, one with the indole nitrogen designated, and one with both nitrogens jointly designated ([:0]NC(=O)c1ccc2[nH]ccc2c1, NC(=O)c1ccc2n([:0])ccc2c1, and [:0]NC(=O)c1ccc2n([:1])ccc2c1). Because in-loop docking is impractical at the generation scale of REINVENT4, the REINVENT4 optimization score was defined as QED + SA_{norm} and used to drive reinforcement learning, and the top 60 molecules by this score were subsequently docked and reranked by QED + SA_{norm} + $Vina_{norm}$. The docking setup was identical to that used for MOZAIC and CReM-dock.
